# Correlation between cortical bone thickness at mini-implant insertion sites and age of patient

**DOI:** 10.1590/2177-6709.27.1.e222098.oar

**Published:** 2022-02-28

**Authors:** Anna Carolina Teixeira CENTENO, Caroline Kolling FENSTERSEIFER, Vitória de Oliveira CHAMI, Eduardo Silveira FERREIRA, Mariana MARQUEZAN, Vilmar Antônio FERRAZZO

**Affiliations:** 1 Universidade Federal de Santa Maria, Curso de Odontologia, Departamento de Estomatologia (Santa Maria/RS, Brazil).; 2 Universidade Federal do Rio Grande do Sul, Faculdade de Odontologia, Departamento de Ortodontia (Porto Alegre/RS, Brazil).

**Keywords:** CBCT, Orthodontic mini-implant, Stability

## Abstract

**Introduction:**

Orthodontic mini-implants (MI) are a reliable alternative to provide temporary orthodontic anchorage. Prior to miniscrew insertion, the best approach would be to evaluate each possible insertion site and measure the cortical bone thickness, and verify whether it would provide adequate primary stability.

**Objective:**

This study aimed to evaluate the difference in cortical bone thickness in areas of mini-implants insertion in patients of different ages, by means of cone beam computed tomography (CBCT).

**Methods:**

The sample of this retrospective study was composed of 123 CBCT scans, which were used to measure cortical bone thickness in the buccal and palatal inter-radicular space in the mesial region of the first permanent molars. These measures were compared by using the Student’s *t*-test, ANOVA/Tukey tests, and Linear regression between male and female subjects, from 12 to 30 years old.

**Results:**

No significant difference was found in cortical bone thickness between sex, race and sagittal facial patterns. Significantly higher measurement values were observed in patients older than 12 years of age at all sites evaluated. The coefficient β at the adjusted linear regression analysis showed that at each increment in age, mean cortical thickness values increased by 0.06mm in the mandible, 0.03mm in the buccal region and 0.02mm in the palatal region of the maxilla.

**Conclusions:**

The increase in cortical bone thickness was positively associated with age; that is, the more advanced the patient’s age was, the less chance there was of failure due to primary stability.

## RESUMO

**Introdução:**

Os mini-implantes ortodônticos (MI) são uma alternativa confiável para fornecer ancoragem esquelética temporária. Antes da inserção do mini-implante, a melhor abordagem seria avaliar cada local de inserção possível, medir a espessura do osso cortical e verificar se proporcionaria uma adequada estabilidade primária.

**Objetivo:**

O presente estudo teve como objetivo avaliar a diferença na espessura do osso cortical em áreas de inserção dos mini-implantes em pacientes de diferentes idades, por meio da tomografia computadorizada de feixe cônico.

**Métodos:**

A amostra desse estudo retrospectivo foi composta por 123 tomografias computadorizadas de feixe cônico, que foram utilizadas para medir a espessura do osso cortical nos espaços inter-radiculares vestibular e palatino na região mesial dos primeiros molares permanentes. Essas medidas foram comparadas por meio dos testes *t* de Student, ANOVA/Tukey e regressão linear entre os sexos masculino e feminino, de 12 a 30 anos.

**Resultados:**

Não houve diferença estatisticamente significativa na espessura cortical, quando comparados sexo, cor da pele e padrão facial sagital. Foram verificadas medidas significativamente maiores em pacientes com idade superior a 12 anos em todos os sítios avaliados. O coeficiente β da análise de regressão linear ajustada mostrou que, a cada incremento da idade, os valores médios da espessura cortical aumentaram 0,06 mm na mandíbula, 0,03 mm na região vestibular e 0,02 mm na região palatina da maxila.

**Conclusão:**

O aumento da espessura do osso cortical teve associação positiva com a idade, ou seja, quanto mais avançada a idade do paciente, menor a chance de falha, devido à estabilidade primária.

## INTRODUCTION

Orthodontic mini-implants (MI) are a reliable alternative to provide temporary orthodontic anchorage.^1^ Approximately 80% of orthodontists use miniscrews, and according to approximately 78% of professionals, they provide better results in orthodontic treatments.^2^ Nevertheless, failure rates range from 11% to 30%.^3^ To stratify the risks of the procedure for inserting anchorage screws, it is necessary to know which site has the thickest cortical bone, at ‘’different ages, for the purpose of guaranteeing the primary stability of the mini-implant and making the outcome of the orthodontic treatment more predictable.

Primary stability of the miniscrews basically depends on the screw design, insertion technique, and quality and quantity of bone at the insertion site.^4^ ^-^ ^7^ Among these factors, the cortical bone thickness of the insertion site is emphasized.^8^ ^,^ ^9^

Prior to miniscrew insertion, the best approach would be to evaluate each possible insertion site by means of cone beam computed tomography (CBCT), to measure the cortical bone thickness, and to verify whether it would provide adequate primary stability. However, using this exam for this purpose is not indicated, according to the American Academy of Oral and Maxillofacial Radiology (AAOMR).^10^

Thus, the aim of this study was to evaluate the cortical bone thickness at different mini-implant insertion sites in the maxilla and mandible, and correlate this with the age of the patient, since the choice of the best site and age has direct repercussion on the reduction of cost and treatment time.

Other variables related to the individuals, such as age, sex, skin color, vertical and sagittal facial patterns would be analyzed through a multivariate analysis, to verify its interaction on the main outcome (age). The hypothesis of the present study was that patients with a more advanced age would present a greater cortical bone thickness, and consequently mini-implants would present a higher degree of primary stability.

## MATERIAL AND METHODS

To conduct this retrospective study, the clinical record charts of patients of the Orthodontic Specialization Course, treated at the Dental School of the Federal University of Rio Grande do Sul (Brazil), were reviewed with regard to the orthodontic documentation of patients that met the following inclusion criteria: (1) initial phase orthodontic patients; (2) mixed or permanent dentition; (3) orthodontic documentation containing data of CBCT performed at the same private radiology center (CBCT scanned in i-CAT^®^, field of view 22x16cm, isotropic voxel of 0.4mm, images acquired with a rotation of 360º during 20s, 120 kVp, 36.90 mAs, and data saved in DICOM format). Patients with pathologies or radiolucency in the areas of measurement; periodontal disease; ectopic eruptions at sites of interest and important medical history were excluded.

The research protocol was submitted to and approved by the Ethics Committee of Federal University of Rio Grande do Sul (CAAE 83140118.4.0000.5347). The database search was performed between July 2017 and April 2018, and 800 orthodontic charts were reviewed. Of these, 123 were selected according to a sample calculation performed with data obtained from a pilot study using 57 individuals from the same database (study power of 80%, significance level of 0.05%, and correlation coefficient of 0.25).

Data regarding age, sex and skin color were collected from the clinical records of the selected patients. In addition, the CBCT images were imported into Dolphin Imaging Cephalometric and Tracing software, version 11.8 (Dolphin Imaging and Management Solutions, Chatsworth, Calif., USA), for the purpose of measuring the cortical bone thickness of the selected sites and assessing the sagittal and the vertical facial patterns. Subjects were categorized according to their facial patterns (sagittal and vertical) using lateral cephalograms (right side) synthesized from the CBCT. For this purpose, the tomographic volume was oriented with the perpendicular sagittal mean plane and the Frankfurt plane (right side of the face) parallel to the ground. Subsequently, the cephalometric analyses of Steiner^11^ and Ricketts^12^ were performed. For classification of the sagittal facial pattern, angle ANB of the Steiner analysis was used: Class I (0° < ANB < 4.5°), Class II (ANB ≥ 4.5°), Class III (ANB ≤ 0°). The vertical facial pattern was determined by means of the Ricketts VERT index, which classifies individuals into dolichocephalic (VERT < -0.50); mesocephalic (-0.49 < VERT < 0.49), and brachycephalic (VERT > 0.50) facial types.

**Figure 1:**
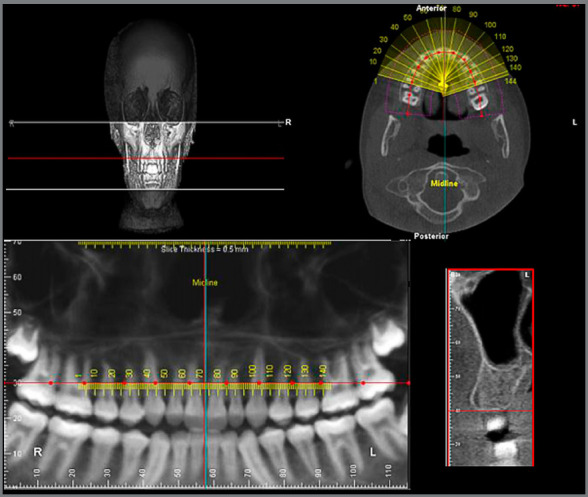
Determination of cross sections.

The cortical bone thickness was measured in cross-sections, generated after determining the arc curvature line (Fig 1). The sites evaluated for cortical bone thickness were: inter-radicular space located in the mesial region of the maxillary and mandibular permanent first molars, where the maxilla was evaluated in the vestibular and palatine regions and the mandible, only in the vestibular region, since these are considered safe sites for mini-implant insertion.^13^ The lingual cortical mandibular bone thickness was not measured, because it is not an area commonly used for miniscrew placement.

**Figure 2:**
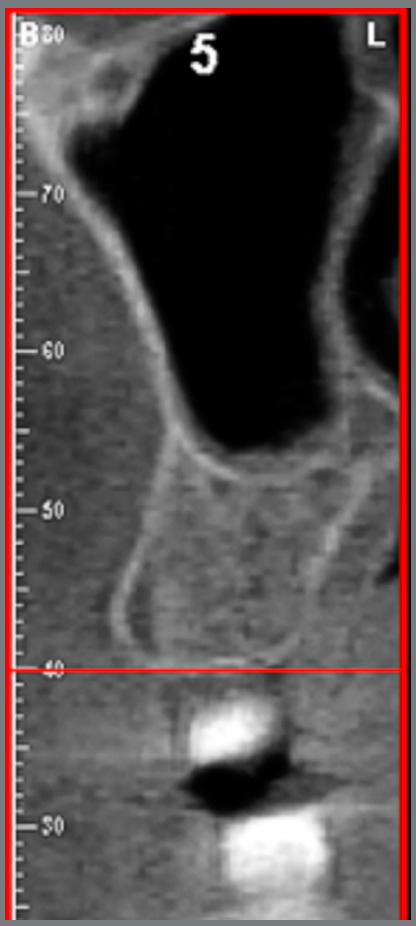
Measurement of vestibular and lingual cortical bone thickness at the determined heights (5mm from the bone crest).

Measurements were performed at a distance of 5 mm from the alveolar bone crest (Fig 2), because there is usually an adequate amount of bone in this position for inserting miniscrews;^14^ ^,^ ^15^ moreover, there is inserted gingiva that favors successful insertion of the device.^16^ For each patient, six measurements (right and left side) were obtained with the millimeter ruler provided by the software. For statistical analysis, the measurements were grouped into three units of evaluation: vestibular maxilla (measurements made on the vestibular cortical bone of the maxilla on both sides); palatal maxilla (measurements made on the palatal bone cortex of the maxilla on both sides); Mandible (measurements made on the vestibular cortical of the mandible on both sides). To minimize possible biases in the survey, each subject was given a registration number, which was obtained by lot, to determine the sequence of the images to be analyzed. A trained and calibrated examiner performed measurements. Twenty-five individuals (20% of the sample) were initially evaluated and reassessed after three weeks to verify reproducibility. The Kappa index was used for categorical variables (ANB and VERT index), and the intraclass correlation coefficient (ICC), for quantitative variables (cortical bone thickness). Excellent reproducibility (Kappa> 0.80 / ICC> 0.75) was found for all measures analyzed.

Statistical analysis was performed in STATA 14.0 software (Stata Corporation, CollegeStation, TX, USA). After a descriptive analysis and normality tests, the Student’s *t-*test was performed to compare means between individuals of the male and female sexes in the age range of 12 years or older, and white and non-white individuals. ANOVA/Tukey tests were performed to compare brachy-, meso- and dolichofacial individuals, and to compare Class I, II and III. Subsequently, unadjusted linear regression analysis was performed to verify the relationship between individual characteristics and cortical bone measurements. After the unadjusted analysis, the variables with *p*-value < 0.20 were included in the adjusted regression analysis. In addition, the sex variable was also included as an adjustment variable. A 95% confidence interval and *p*-value < 0.05 represented a statistically significant relationship.

## RESULTS

The median age of the 123 patients selected was 12.1 years (7.6 - 30.7). Table 1 shows the sample frequencies and distribution by sex, age, skin color, vertical facial pattern (VERT) and sagittal facial pattern (ANB).

**Table 1:** Descriptive distribution of sex, age, skin color and facial patterns of the sample.

	n (%)
Sex	
Male	53 (43.09)
Female	70 (56.91)
Age	
< 12 years	64 (52.03)
>12 years	59 (47.97)
Skin color	
White	107 (86.99)
Non-white	16 (13.01)
VERT	
Dolichofacial	28 (22.76)
Mesofacial	42 (34.15)
Brachyfacial	53 (43.09)
ANB	
Class I	43 (34.96)
Class II	61 (49.59)
Class III	19 (15.45)

Unadjusted and adjusted linear regression analysis for sex, skin color, age, vertical facial (VERT) and sagittal (ANB) facial pattern was performed to simultaneously evaluate the effect of multiple individual patient factors on the cortical bone thickness (Table 2). The results of adjusted linear regression analysis showed that sex, skin color and sagittal facial pattern had no significant effect on bone thickness. However, the increase in age significantly influenced the increase in cortical bone thickness in all evaluated areas. The coefficient β showed that with each increase in age, the mean cortical thickness values increased by 0.06mm in the mandible, 0.03mm in the vestibular region of the maxilla and 0.02mm in the palatal region of the maxilla. The direction of facial growth was shown to influence the thickness of the mandibular vestibular cortical bone (*p*< 0.00).

**Table 2:** Comparison between individual characteristics and bone measurements (Student *t*-test and ANOVA/Tukey).

	Mandible Mean (SD)	***p*-value**	Vestibular Maxilla Mean (SD)	***p*-value**	Palatal Maxilla Mean (SD)	***p*-value**
Sex		0.82		0.17		0.23
Male	2.65 (0.64)		1.94 (0.36)		2.05 (0.34)	
Female	2.62 (0.60)		2.03 (0.32)		2.12 (0.33)	
Age		<0.00		<0.00		<0.00
< 12 years	2.44 (0.51)		1.87 (0.27)		1.98 (0.30)	
>12 years	2.84 (0.65)		2.12 (0.36)		2.20 (0.34)	
Skin color		0.70		0.39		0.22
White	2.63 (0.63)		1.98 (0.35)		2.08 (0.35)	
Non-white	2.68 (0.50)		2.05 (0.26)		2.16 (0.23)	
VERT		0.02*		0.38		0.47
Dolichofacial	2.41 (0.48) ^a^		1.96 (0.35)		2.02 (0.36)	
Mesofacial	2.59 (0.59) ^a,b^		1.95 (0.35)		2.09 (0.32)	
Brachyfacial	2.79 (0.67) ^b^		2.04 (0.33)		2.12 (0.34)	
ANB		0.20		0.22		0.15
Class I	2.76 (0.74)		2.06 (0.33)		2.15 (0.32)	
Class II	2.55 (0.55)		1.94 (0.33)		2.02 (0.31)	
Class III	2.61 (0.45)		2.04 (0.35)		2.15 (0.38)	

* Statistically significant difference.

Different letters represent statistically significant differences between groups (*p*< 0.05).

Significantly higher mean cortical bone thickness values were observed in patients over 12 years of age in all the evaluated sites. As regards the vertical facial pattern, brachycephalic patients had the highest mean cortical bone thickness values in all evaluated areas, but significant differences were observed only in the mandibular vestibular cortical bone (Table 3). More information about the patients of the sample (gender, race and age) can be found at the appendix A.

**Table 3:** Unadjusted and adjusted linear regression analysis between the individual characteristics and the cortical bone measurements.

	Mandible Unadjusted regression		Mandible Adjusted regression	
	β coefficient (CI-95%)	***p*-value**	β coefficient (CI-95%)	***p*-value**
Sex		0.82		
Male	1		-	
Female	-0.03 (-0.25-0.20)		-	
Skin color		0.74		
White	1		-	
Non-white	0.05 (-0.27-0.38)		-	
Age	0.06 (0.04-0.09)	<0.00	0.06 (0.04-0.08)	<0.00
VERT	0.18 (0.07-0.29)	<0.00	0.15 (0.06-0.25)	<0.00
ANB	0.02 (-0.19-0.06)	0.29	-	
	Vestibular Maxilla Unadjusted regression		Vestibular Maxilla Adjusted regression	
	β coefficient (CI-95%)	*p*-value	β coefficient (CI-95%)	*p*-value
Sex		0.16		0.30
Male	1		1	
Female	0.09 (-0.03-0.20)		0.06 (-0.05-0.17)	
Skin color				
White	1	0.48	-	
Non-white	0.06 (-0.11-0.24)		-	
Age	0.03 (0.02-0.04)	<0.00	0.03 (0.01-0.04)	<0.00
VERT	0.06 (-0.00-0.12)	0.07	0.03 (-0.02-0.09)	0.23
ANB	-0.00 (-.02-0.02)	0.98	-	
	Palatal Maxilla Unadjusted regression		Palatal Maxilla Adjusted regression	
	β coefficient (CI-95%)	*p*-value	β coefficient (CI-95%)	*p*-value
Sex		0.23		
Male	1		-	
Female	0.07(-0.05-0.19)		-	
Skin color		0.36		
White	1		-	
Non-white	0.08 (-0.09-0.26)		-	
Age	0.02 (0.01-0.03)	<0.00	0.02 (0.01-0.03)	<0.00
VERT	0.03 (-0.03-0.09)	0.35	-	
ANB	0.01 (-0.01-0.03)	0.42	-	

## DISCUSSION

The success of miniscrews is related to their primary stability that is defined by the absence of mobility in the bone after their insertion^17^ and this depends on the adaptation and mechanical retention of these devices in the bone tissue.^18^ The anatomy of the bony site, especially the cortical bone thickness, plays a fundamental role in this mechanical bracing, and consequently influences the success or failure of the device.

The results of this study suggested that the cortical bone thickness varied according to the age of the individuals. Young patients tended to have thinner cortical bone, in comparison with older individuals. Brachycephalic patients tended to have thicker cortical bone in the mandible. The variables sex, skin color, and sagittal facial pattern did not significantly influence the cortical bone thickness of the mandible and maxilla.

The difference in cortical bone thickness found in patients of different age groups may explain the results obtained in previous studies that observed the rate of maxillary miniscrews loss in adolescents. Moon et al.^19^, in a clinical study that evaluated the influencing factors and success rate of 778 miniscrews in 306 patients, reported success in 76.1% of adolescents and 87.3% of adults. The increase in cortical bone thickness could be due to changes in the functional capacity of the individuals, since the masticatory force, muscle size and activity tend to increase with age.^20^ ^-^ ^22^

The results obtained corroborated the findings of previous studies that evaluated the cortical thickness at miniscrew insertion sites. Farnsworth et al.^23^ correlated the cortical thickness with the age and sex of patients. However, they found thicker cortical bone in adults (20-45 years) when compared with adolescents (11-16 years).

Ohiomoba et al.^24^ showed that the increase in age was positively correlated with cortical bone thickness: 16 year-old or older patients had significantly thicker cortical bone, in comparison with patients between 12 and 16 years of age, but the bone thickness remained almost constant from the age of 16 years onwards. Similar results were reported by Fayed et al.^25^: individuals between 19 and 27 years of age showed thicker vestibular and palatal cortical bone when compared with the younger patients (13-18 years).

In the present study, brachycephalic patients showed thicker cortical bone, both in the maxilla and mandible, when compared with mesocephalic and dolichocephalic patients, however, this difference was statistically significant only in the mandibular cortical bone. Swasty et al.^26^ observed the same differences in vertical facial patterns.

The linear regression results showed that the vertical facial pattern influenced the mandibular cortical measurements. This tendency was observed in the vestibular cortical of the maxilla, however, without statistical significance. This result may have been due to the real absence of association or due to sample size. Horner et al.^27^ evaluated the cortical bone thickness in hyperdivergent and hypodivergent adults and concluded that in the majority of the studied sites, hypodivergent patients presented thicker cortical bone than hyperdivergent individuals.

Although the data showed that the cortical thickness values of non-white patients were higher than those of white individuals in all the evaluated sites, this difference was not statistically significant; and in the multivariate regression the skin color showed no influence on the cortical thickness. However, further studies are needed with groups that have a balanced distribution, considering that the sample of the present study included 107 white individuals and only 16 non-white individuals.

In addition, it is suggested that other variables should be included in future studies such as: the individuals’ diet and masticatory force, since they are factors that may be associated with the difference in bone thickness and density. Clinical studies are also encouraged to evaluate the success rate of miniscrews and the variables that may influence the bone characteristics of patients.

A limitation of the present study was the 0.4 mm voxel size used in the CBCT acquisition protocol. According to Ballrick et al.^28^, the mean spatial resolution for voxel used in orthodontics is 0.7 mm. Thus, the accuracy of measurements smaller than 0.7 mm was not reliable, and should be observed with caution. However, considering that the advantage would be an increase in the precision and accuracy of the measurements obtained by means of CBCT, the disadvantage produced by the reduction in voxel size from 0.4mm to 0.25mm would be the increase in the dose of ionizing radiation to which the patients would be exposed. Moreover, the minority of thickness values obtained in this study were lower than 0.7 mm.

The main contribution of this study was the evaluation of cortical bone thickness with reference to different variables related to the individuals, such as age, sex, skin color and vertical and sagittal facial patterns, by performing the multivariate analysis, which made it possible to verify the influence of each individual variable and its interaction on the outcome.

It is important to point out that although young patients (<12 years) are not the individuals commonly eligible for the placement of miniscrews, their inclusion in this study allowed the variation in the pattern of cortical bone thickness to be according to age.

## CONCLUSIONS

The increase of cortical bone thickness was positively associated with age. Adjusted linear regression analysis showed that at each increase in age, the mean cortical thickness values increased by 0.06mm in the mandible, 0.03mm in the vestibular region and 0.02mm in the palatal region of the maxilla. Brachycephalic patients presented higher cortical bone thickness values. The variables sex, sagittal facial pattern and skin color of the patients did not influence the cortical bone thickness in the interradicular areas of miniscrews insertion.

## Appendix A: All patients gender, race and age.

**Table:**

Patient	Gender	Race	Age
1	F	W	12y 5m
2	F	W	12y 10m
3	F	W	30y
4	F	W	13y 8m
5	M	W	27y 10m
6	F	W	12y 3m
7	F	W	12y 8m
8	M	W	14 y 1m
9	M	W	13y 8m
10	M	W	17y
11	F	W	17y 3m
12	F	W	18y 9m
13	F	W	18y 10m
14	F	W	16y 4m
15	M	W	15y 6m
16	F	NW	12y 10m
17	F	W	16y 10m
18	F	W	11y 7m
19	F	W	14y 5m
20	F	W	11y 7m
21	F	NW	26y 5m
22	M	W	12y 10m
23	F	W	30y 7m
24	M	W	13y 6m
25	F	W	12y 7m
26	M	W	13y 7m
27	M	W	15y 3m
28	M	W	13y 5m
29	M	W	13y 2 m
30	M	W	13y 6m
31	M	W	15y 3m
32	F	NW	16y 7m
33	M	NW	13y 10m
34	F	W	13y 5m
35	M	W	14y 9m
36	M	W	12y 11m
37	F	W	15y 6m
38	F	NW	11y 2m
39	M	NW	16y3m
40	F	NW	21y
41	F	W	14y 5m
42	F	W	11y 2m
43	F	W	12y11m
44	M	NW	22y 10m
45	M	W	16y
46	M	W	12y4m
47	F	W	17y 2m
48	F	W	26y4m
49	F	W	14y3m
50	F	W	12y8m
51	F	W	12y11m
52	M	W	13y1m
53	F	W	16y6m
54	F	NW	12y2m
55	F	NW	12y6m
56	M	W	25y8m
57	M	W	14y2m
58	M	W	16y4m
59	F	W	23y6m
60	F	W	26y4m
61	M	NW	11y 4m
62	M	W	19y10m
63	F	W	11y11m
64	F	W	13y7m
65	M	W	11y
66	F	W	12y11m
67	M	W	12y1m
68	M	NW	17y2m
69	F	W	10y10m
70	M	W	12y7m
71	F	W	12y5m
72	F	W	11y8m
73	F	W	10y5m
74	F	W	13y7m
75	F	W	13y10m
76	F	W	10y
77	F	W	11y5m
78	M	W	14y6m
79	F	W	10y7m
80	F	W	11y11m
81	F	W	15y1m
82	M	W	11y7m
83	F	W	15y6m
84	F	W	14y 6m
85	F	NW	30y2m
86	M	W	11y4m
87	F	W	10y11m
88	F	W	11y2m
89	F	W	9y9m
90	M	W	10y9m
91	F	W	10y9m
92	M	W	9y11m
93	M	W	13y2m
94	F	W	13y1m
95	M	W	11y6m
96	F	W	9y1m
97	M	W	9y5m
98	M	W	8y10m
99	M	W	9y7m
100	F	W	7y6m
101	F	W	10y5m
102	M	W	12y5m
103	F	W	10y8m
104	M	W	12y6m
105	M	NW	11y11m
106	M	W	12y10m
107	M	W	7y11m
108	F	W	12y3m
109	M	W	11y1m
110	M	W	9y10m
111	F	W	10y7m
112	F	NW	10y10m
113	M	W	10y4m
114	F	W	11y4m
115	M	W	11y4m
116	M	W	8y3m
117	F	W	8y8m
118	F	W	8y2m
119	M	W	10y2m
120	F	NW	8y6m
121	M	W	12y
122	M	W	14y7m
123	F	W	18y1m

NW= Non-white.
